# Supplementary material for: A tripartite organelle platform links growth factor receptor signaling to mitochondrial metabolism
Source: Nat Commun. 2024 Jun 15;15:5119. doi: 10.1038/s41467-024-49543-z (PMC11180189; doi:10.1038/s41467-024-49543-z)
Supplement: Supplementary file 1 — Supplementary Information [file 41467_2024_49543_MOESM1_ESM.pdf]

Supplementary Information

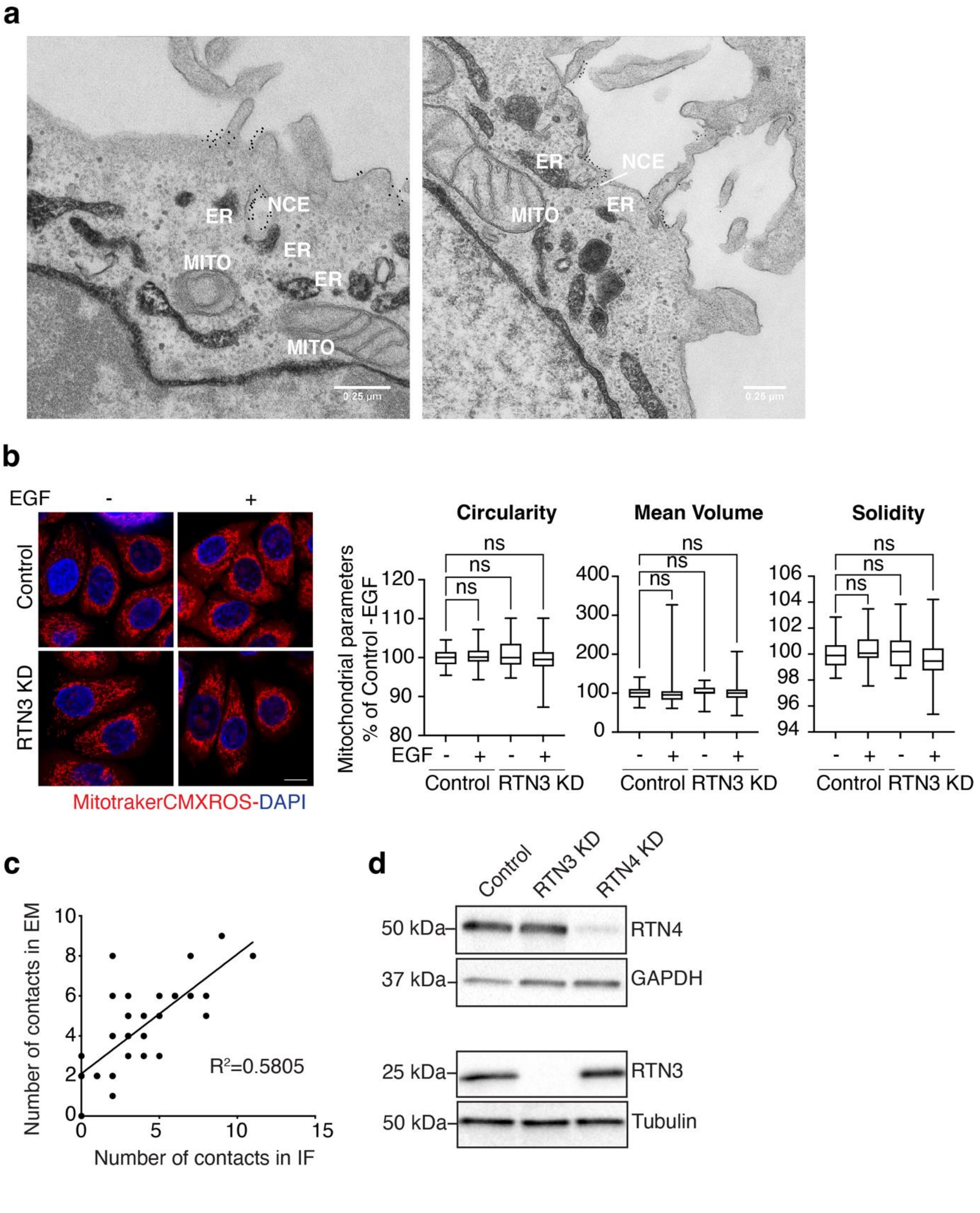

Figure S1

Fig. S1. EGFR-NCE involves tripartite PM-ER-mitochondria contact sites, related to Fig. 1. a Immuno-EM micrographs showing the close spatial relationship between NCE tubules

(CD147-10 nm gold), the ER (HRP-KDEL/DAB), and mitochondria in HeLa cells treated with high dose EGF for 5 mins. Bars 0.25 μM. **b** HeLa cells were either subjected to RTN3 KD or left untreated, followed by *in vivo* labelling with Mitotracker CMXROS for 30 minutes at 37°C in complete medium. After two washes with PBS, cells were stimulated with high-dose EGF (100 ng/ml), for 5 minutes at 37°C. Left, representative confocal images are shown. Blue, DAPI. Bar, 10 μm. Right, Quantification of mitochondria morphology parameters (circularity, mean volume, and solidity) via an *ad hoc* macro on ImageJ. Each parameter is reported as a percentage relative to unstimulated control. N, number of cells: Control -EGF/N=539, Control +EGF/N=524, RTN3-KD -EGF/N=400, RTN3-KD +EGF/N=364 (n=2). **c** Correlation analysis of ER-mitochondria contacts on CLEM images. For each CLEM image pair, the number of contacts revealed by ImageJ JACoP plugin in the fluorescent image was plotted against the number of ER-mitochondria contacts (<20nm) observed in the corresponding EM image. R^2 = correlation coefficient. **d** Efficiency of RTN3 and RTN4 KD in HeLa cells was analyzed by IB. Control cells were mock treated. GAPDH, loading control. MW markers are shown on the left. In the box plots, the lower and upper boundaries of the box are the first and third quartiles, with the median annotated with a line inside the box. The whiskers extend to the maximum and minimum values. All panels, P-value (Each Pair Student's t-test, two-tailed): *, <0.05; **, <0.01; ***, <0.001, ns, not significant; n=biological replicates. Source data are provided as a Source Data file.

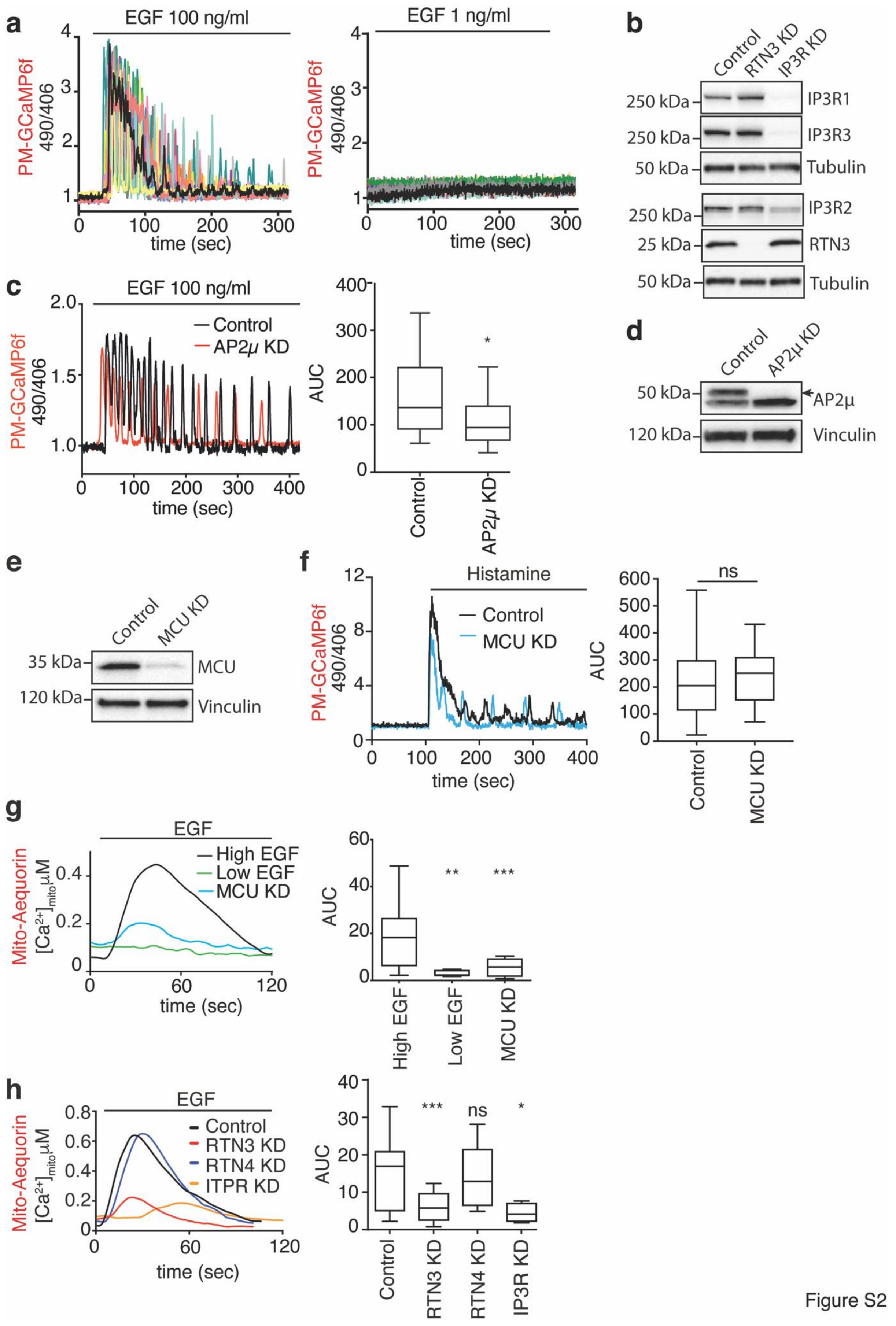
a
EGF 100 ng/ml
EGF 1 ng/ml
PM-GCaMP6f 490/406
time (sec)
b
Control
RTN3 KD
IP3R KD
250 kDa
IP3R1
250 kDa
IP3R3
50 kDa
Tubulin
250 kDa
IP3R2
25 kDa
RTN3
50 kDa
Tubulin
c
EGF 100 ng/ml
Control
AP2μ KD
PM-GCaMP6f 490/406
time (sec)
AUC
*
Control
AP2μ KD
d
Control
AP2μ KD
50 kDa
AP2μ
120 kDa
Vinculin
e
Control
MCU KD
35 kDa
MCU
120 kDa
Vinculin
f
Histamine
Control
MCU KD
PM-GCaMP6f 490/406
time (sec)
AUC
ns
Control
MCU KD
g
EGF
High EGF
Low EGF
MCU KD
Mito-Aequorin [Ca2+]mito μM
time (sec)
AUC
**
***
High EGF
Low EGF
MCU KD
h
EGF
Control
RTN3 KD
RTN4 KD
ITPR KD
Mito-Aequorin [Ca2+]mito μM
time (sec)
AUC
***
ns
*
Control
RTN3 KD
RTN4 KD
IP3R KD

25

Figure S2

Fig. S2. EGF-induced Ca^{2+} response at the PM and inside mitochondria, related to Fig. 2. **a** HeLa PM-GCaMP6f cells were stimulated with low or high dose EGF. The Ca^{2+} response was monitored by measuring fluorescence over time. Results are presented as the ratio of the emission at 490/406 nm for individual cells (represented by different colors). **b** Efficiency of RTN3 KD and IP3R KD (IP3-R1, IP3-R2, IP3-R3) in HeLa cells was analyzed by IB. Tubulin, loading control. MW markers shown on the left. Samples have been run and blotted on two independent gels. IP3R3 has been reblotted after IP3R1, after secondary antibody inactivation with sodium azide. **c** The Ca^{2+} response was measured in HeLa cells subjected or not to AP2µ KD and stimulated with high EGF as in (a). Results are presented as the ratio of the emission at 490/406 nm. Left, representative single cells traces. Right, AUC is reported. Control/High EGF/N=18, AP2µ-KD/High/EGF/N=18, N=number of cells (n=1). **d** Efficiency of AP2µ KD in HeLa cells analyzed by IB (the arrow indicates the specific bands). Vinculin, loading control. MW markers shown on the left. **e-f** Additional controls for main Fig. 2C. (e) Efficiency of MCU KD in HeLa cells analyzed by IB. Vinculin, loading control. MW markers shown on the left. (f) Histamine induces a Ca^{2+} response independently of MCU. HeLa PM-GCaMP6f cells were subjected to MCU KD and stimulated with histamine (100 µM). The kinetics of the Ca^{2+} response was monitored by measuring fluorescence. Results are presented as (c). Control/Histamine/N=91, MCU KD/Histamine/N=85, a representative experiment out of two independent replicates is shown. **g** HeLa cells expressing Aequorin targeted to the mitochondria (Mito-Aequorin) were stimulated with low EGF or high EGF alone or with MCU KD. Ca^{2+} waves inside mitochondria were detected by luminescence. Left, representative traces. Right, AUC is reported. Low EGF/N=6/n=3; High EGF/N=29/n=9; High EGF/MCU KD/N=15/n=5. N=number of coverslips (whole cell population). **h** HeLa cells expressing Mito-Aequorin were subjected or not to the indicated KDs and stimulated with high EGF (100 ng/ml), during luminescence recording. Results are presented as in (f). Control/HighEGF/N=25/n=7; RTN3-KD/HighEGF/N=16/n=5; RTN4-KD/HighEGF/N=11/n=5; IP3R-KD/HighEGF/N=4/n=2. In the box plots, the lower and upper boundaries of the box are the first and third quartiles, with the median annotated with a line inside the box. The whiskers extend to the maximum and minimum values. All panels, P-value (Each Pair Student's t-test, two-tailed): *, <0.05; **, <0.01; ***, <0.001, ns, not significant; n=biological replicates. Source data are provided as a Source Data file.

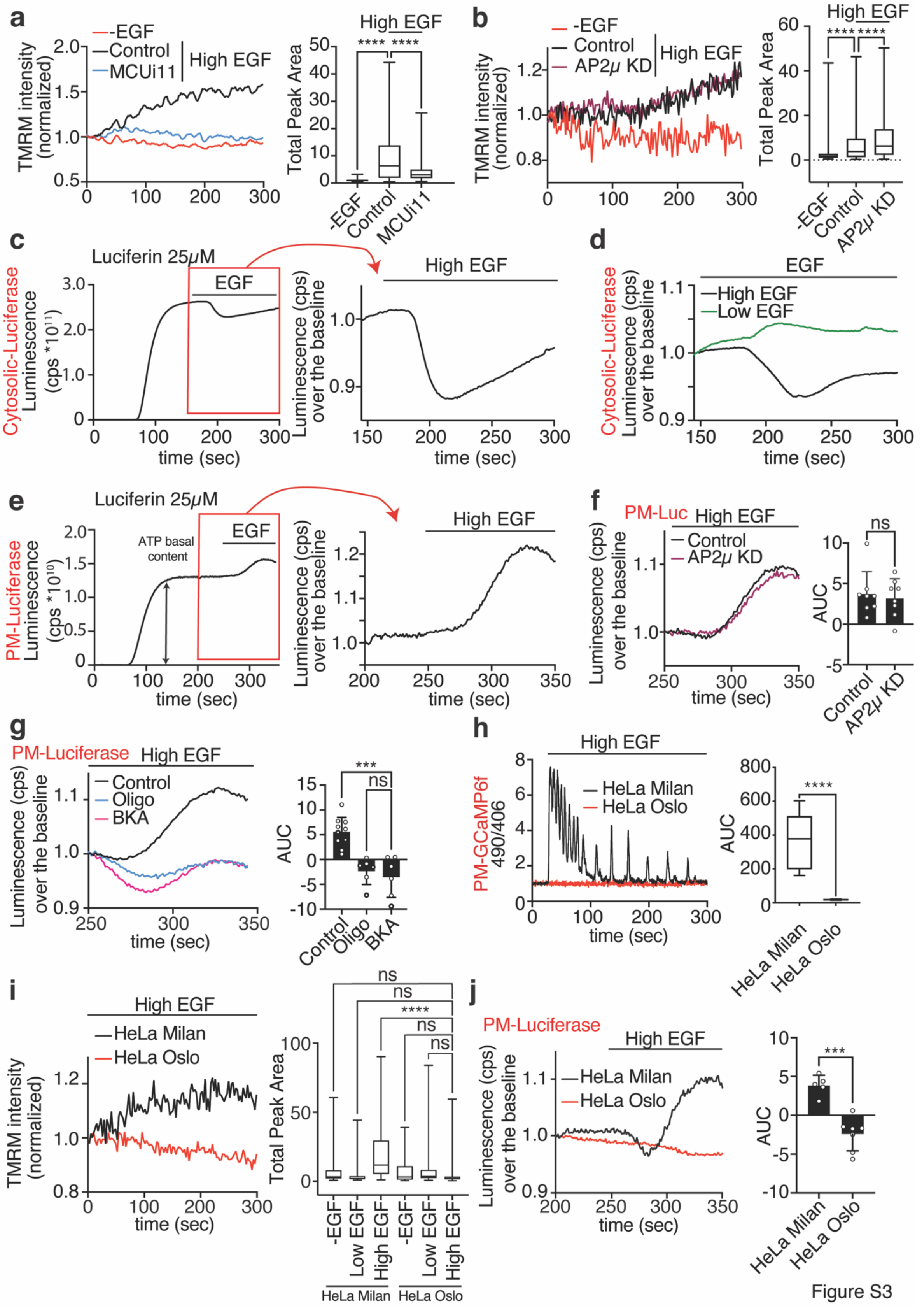

Fig. S3. Characterization and specificity of EGF-induced Ca^{2+} oscillations and mitochondrial ATP production, related to Fig. 3. a HeLa cells were pre-treated with MCU

inhibitor (MCUi11, 50 µM) or vehicle control for 30 min, then labeled with TMRM and either left unstimulated (-EGF) or stimulated high EGF (100 ng/ml). Fluorescence was recorded over 5 min. Left, fluorescence TMRM intensity. Right, Total peak area. -EGF/N=50, Control/High EGF/N=139, MCUi11/High EGF/N=132, N=number of cells (n=1). **b** TMRM fluorescence measured in HeLa cells, subjected to AP2µ KD or mock control and stimulated high dose EGF for 5 min. Unstimulated mock cells (-EGF) served as the negative control. Left, time course of TMRM fluorescence intensity. Right, Total peak area. -EGF/N=155 (n=1), Control/N=822, AP2µ-KD/N=782, N=number of cells; n=3. Note that AP2 KD caused an increase in TMRM fluorescence relative to the high EGF control. **c** HeLa cells expressing cytosolic-luciferase were incubated with 25 µM luciferin and basal luminescence was recorded (representing an estimation of the basal ATP level in the cytosol). EGF was perfused after 250 s and reached cells after approximately 30 sec. Left, representative complete luminescence time course. Right, magnification of the red boxed area, showing luminescence levels over the baseline just before and after the addition of EGF; cps, count per seconds. **d** HeLa cells expressing cytosolic-luciferase were treated as in (c) and stimulated with low and high dose EGF. Representative curves of luminescence over the baseline after the addition of EGF are shown. Low EGF/N=8, High EGF/N=19, N=number of coverslips (whole cell population). **e** HeLa cells expressing PM-Luc were incubated with 25 µM luciferin and basal luminescence was recorded. EGF (high dose) was perfused after 250 s and reached cells after approximately 30 sec. Left, representative complete luminescence time course. Right, magnification of the red boxed area. **f** Luminescence was measured in HeLa cells expressing PM-luc and subjected or not to AP2µ KD. Cells were treated with 25 µM luciferin. EGF (high dose) was perfused after 250 s and reached cells after approximately 30 sec. Left, representative curves of luminescence over the baseline are shown. Right, mean AUC ±SD. Control/N=8, AP2µ KD/N=8, N=number of coverslips; n=2. **g** HeLa cells expressing PM-Luc were treated with bongkrekic acid (BKA, 50 µM) for 15 mins before addition of 25 µM luciferin and luminescence recording. EGF (high dose) was perfused after 250 s and reached cells after approximately 30 sec. Note that data reported here for Oligomycin (OMY) and Control are the same as in Fig. 3h. Left, representative curves of luminescence over the baseline. Right, mean AUC ±SD. Control/N=10, BKA/N=6, OMY/N=6, N=number of coverslips (whole cell population); n=2, **h-j** HeLa Oslo cells, a clone lacking NCE but has CME, is unable to activate an EGF-dependent Ca^{2+} signaling at the PM and the mitochondrial response. (h) HeLa Milan (clone used in experiments throughout) and HeLa Oslo cells expressing PM-GCaMP6f were stimulated with

high dose EGF. The kinetics of the Ca^{2+} response was monitored by measuring fluorescence. Results are presented as the ratio of the emission at 490/406 nm. Left, representative single cell response curves. Right, AUC is reported. Milan/N=12, Oslo/N=12, N=number of cells; a representative experiment of n=3 is shown. (i) HeLa Milan and Oslo cells labeled with TMRM were left unstimulated (-EGF) or stimulated with low or high EGF and fluorescence recorded. Left, representative time course of fluorescence intensity for cells stimulated with high EGF. Right, total peak area. HeLa Milan: -EGF/N=165, Low EGF/N=147, High EGF/N=322. HeLa Oslo: -EGF/N=39, Low EGF/N=173, High EGF/N=146, N=number of cells; a representative experiment of n=2 is shown. (j) Luminescence was recorded in HeLa Milan and Oslo cells expressing PM-Luc and treated with 25 μM luciferin. EGF (high dose) was perfused after 250 s and reached cells after approximately 30 sec. Left, representative curves of luminescence over the baseline before and after the addition of EGF are shown. Right, mean AUC ±SD. HeLa Milan/N=5, HeLa Oslo/N=7 (n=1). In the box plots, the lower and upper boundaries of the box are the first and third quartiles, with the median annotated with a line inside the box. The whiskers extend to the maximum and minimum values. All panels, P-value (Each Pair Student’s t-test, two-tailed): ***, <0.001, ****, <0.0001, ns, not significant; n=biological replicates. Source data are provided as a Source Data file.

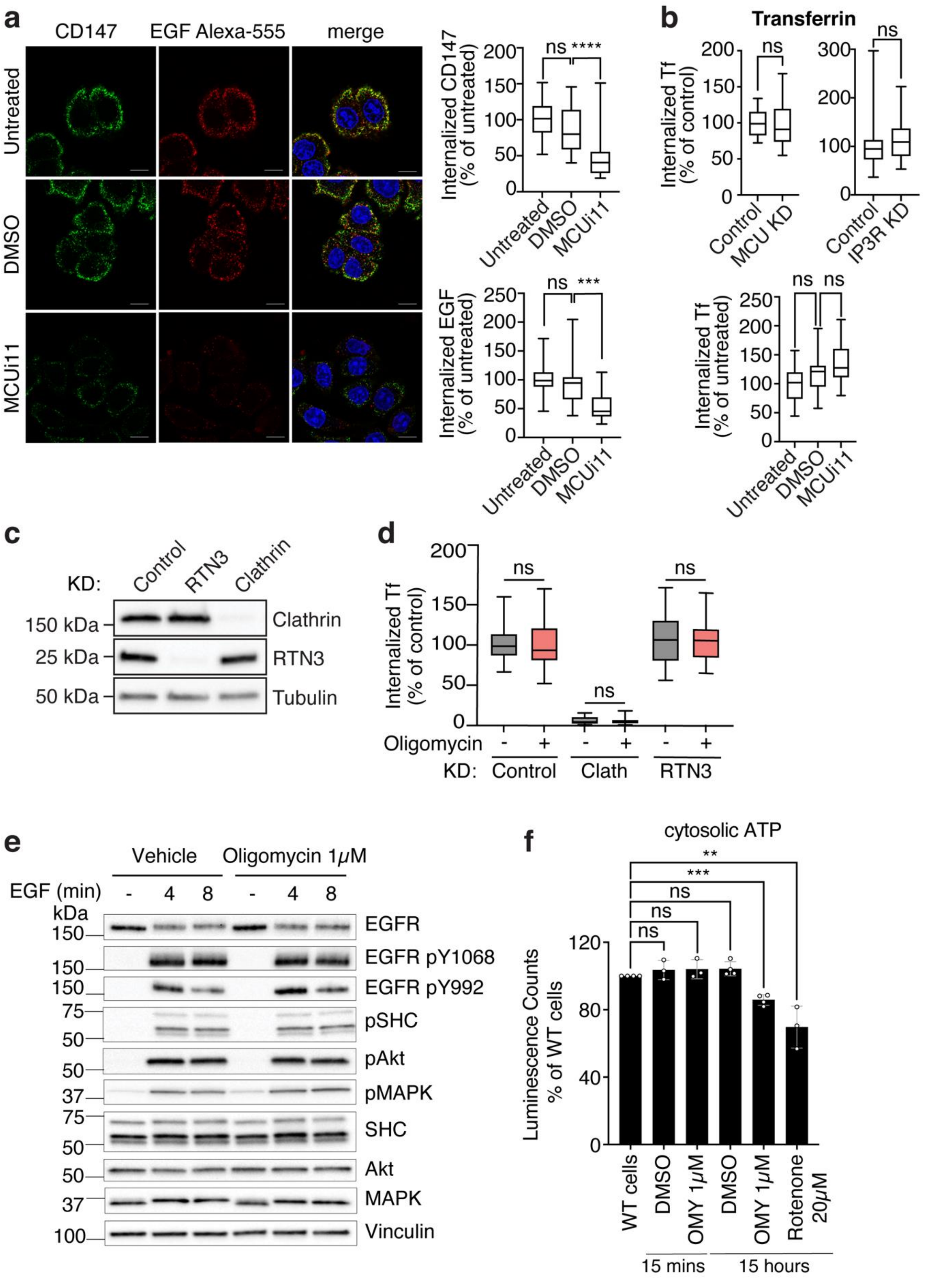

Fig. S4. EGF-induced Ca^{2+} waves and mitochondrial ATP production are required for NCE execution, related to Fig. 5. a HeLa cells treated with MCUi11 (50 μM for 30 min),

DMSO vehicle control or left untreated, were incubated *in vivo* with a specific anti-CD147 antibody for 60 min at 4°C, then with an Alexa-488 secondary antibody (green) for 30 min at 4°C and then stimulated with high dose Alexa647-EGF (100 ng/ml, red) for 5 min at 37°C. Cells were subjected to acid wash treatment prior to fixation to visualize only internalized CD147/EGF. MCUi11 was kept during stimulation. Blue, DAPI. Bar, 10 µm. Right, Quantification of internalized CD147 (top) and EGF (bottom). Mean integrated fluorescence intensity is reported as a percentage relative to control. N=number of cells: untreated/N=116, DMSO/N=110, MCUi11/N=115 (n=2). **b** Transferrin (Tf) internalization in HeLa cells subjected to the indicated KDs and treatments. Alexa647-Tf internalization was followed for 8 min at 37°C in HeLa cells. Mean integrated fluorescence intensity is reported as a percentage relative to control. N=number of cells: Control/N=115, MCU-KD/N=96 (n=2); Control/N=396, IP3R-KD/N=324 (n=4); untreated/N=138, DMSO/N=138, MCUi11/N=139 (n=2). **c** Efficiency of RTN3 and clathrin KD in HeLa cells, analyzed by IB. Tubulin, loading control. MW markers shown on the left. **d** Transferrin (Tf) internalization in HeLa cells subjected to OMY treatment or RTN3 KD or clathrin KD was in measured and reported as in (a). N=number of cells: Control/N=244, Control+OMY/N=250 (n=4); Clathrin-KD/N=231, Clathrin-KD+OMY/N=209, (n=4); RTN3-KD/N=185, RTN3-KD+OMY/N=183 (n=3). **e** IB analysis of the expression and phosphorylation status of EGFR and the indicated signaling effectors in HeLa cells, treated with OMY (1 µM, pretreatment for 5 min, as in main Figs. 4b, d) or vehicle and stimulated or not with high EGF dose (100 ng/ml) for different timepoints. Vinculin, loading control. MW markers shown on the left. Samples have been run and blotted on four independent gels. A representative loading control (vinculin) is shown. Note that acute OMY treatment does not affect EGFR activation nor its downstream signaling, suggesting that the inhibition of NCE is independent of EGFR activation. **f** Measurement of the total cellular ATP content (measured with CellTiter-Glo, G7570, Promega) upon acute treatment with OMY (1 µM, pretreatment for 5 min). Chronic treatment with either OMY (1 µM for 15 h) or rotenone (20 µM for 15 h) was used as a positive control of cytosolic ATP reduction. Note that acute OMY treatment does not affect the total ATP content, while it affects the local burst of ATP induced by EGF (**Fig. 3h**) and EGF/CD147 NCE (**Fig. 5b**). Mean luminescence ±SD is reported as a percentage relative to WT cells. For each condition, n=3, except for WT, DMSO and OMY 1 µM for 15 hours n=4. In the box plots, the lower and upper boundaries of the box are the first and third quartiles, with the median annotated with a line inside the box. The whiskers extend to the maximum and minimum values. All panels, P-value (Each Pair

Student’s t-test, two-tailed): *, <0.05; **, <0.01; ***, <0.001, ****, <0.0001, ns, not significant; n=biological replicates. Source data are provided as a Source Data file.

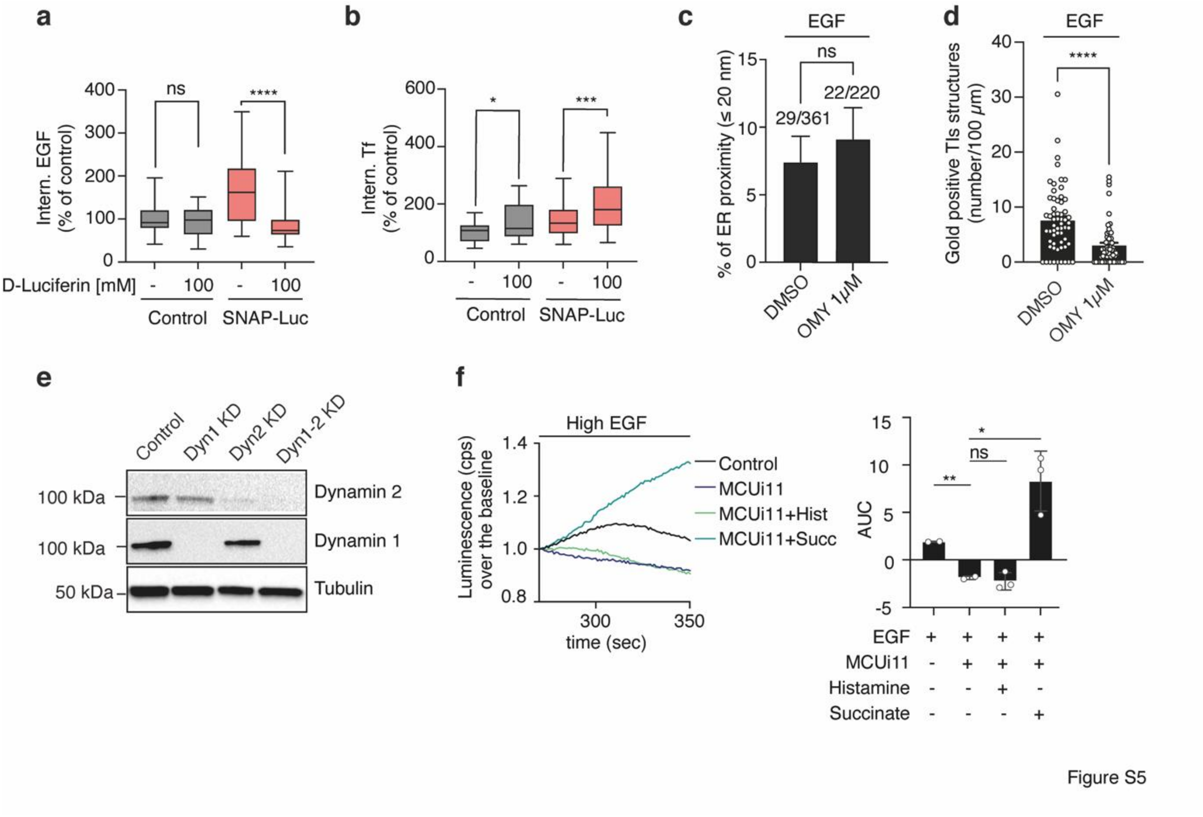

Fig. S5. EGF-induced Ca^{2+} waves and mitochondrial ATP production are required for NCE execution, related to Fig. 5. a, b HeLa cells were transfected with PM-Luc and treated or not with luciferin (100 µM). Internalization of EGF (a) or Tf (b) was monitored for 8 min at 37°C. Relative EGF/Tf fluorescence intensity is expressed as % of control. N, number of cells for EGF: Control/N=372; Control+Luciferin/N=354; SNAP-Luc/N=190; SNAP-Luc+Luciferin/N=185 (n=6). N, number of cells for Tf: Control/N=258 (n=4); Control+Luciferin/N=270 (n=4); SNAP-Luc/N=129 (n=4); SNAP-Luc+Luciferin/N=136 (n=4). **c** Mean frequency of ER proximity with gold EGFR–labelled TIs in HeLa cells either left untreated or pretreated with OMY (1 µM for 5 mins), and stimulated with high dose EGF for 5 min (in the presence or not of the drug). Results are expressed as percentage ± SEM. The number of counted structures in contact with the ER is indicated (distance ≤ 20 nm). N= number of cell profiles analyzed. DMSO/N=20, OMY/N=20. **d** Quantification of gold-labeled EGFR-positive TIs in HeLa cells treated as in (c) and stained with Ruthenium red (RuR). Only RuR-stained structures were counted. Data are expressed as the number of gold-positive structures, normalized to PM profiles of 100-µm length, ±SEM. N, cell profiles analyzed, DMSO/N=60,

OMY/N=56 (n=3). **e** Efficiency of Dynamin 1 and Dynamin 2 KDs in HeLa cells (used in Fig. 5D, E), as compared to mock-treated control, analyzed by IB. Tubulin, loading control. MW markers shown on the left. Dynamin 2 has been reblotted after Dynamin 1, after secondary antibody inactivation with sodium azide. **f** Additional controls for main Fig. 5F. Succinate treatment rescues the PM-localized ATP increase induced by high EGF in MCU inhibited cells. HeLa cells expressing PM-Luc were left untreated or treated with MCUi11 (50 µM for 30 mins and kept during the recording) alone or in combination with histamine (100 µM) or succinate (5 mM), and then were stimulated with high EGF (100 ng/ml), during luminescence recording. Left, representative curves of luminescence over the baseline after the addition of EGF; cps, count per second. Right, mean AUC ±SD. N=number of coverslips (whole cell population): Control/N=2, MCUi11/N=2, MCUi11/Histamine/N=3, MCUi11/Succinate/N=3 (n=1). In the box plots, the lower and upper boundaries of the box are the first and third quartiles, with the median annotated with a line inside the box. The whiskers extend to the maximum and minimum values. All panels, P-value (Each Pair Student's t-test, two-tailed): *, <0.05; **, <0.01; ***, <0.001, ns, not significant; n=biological replicates. Source data are provided as a Source Data file.

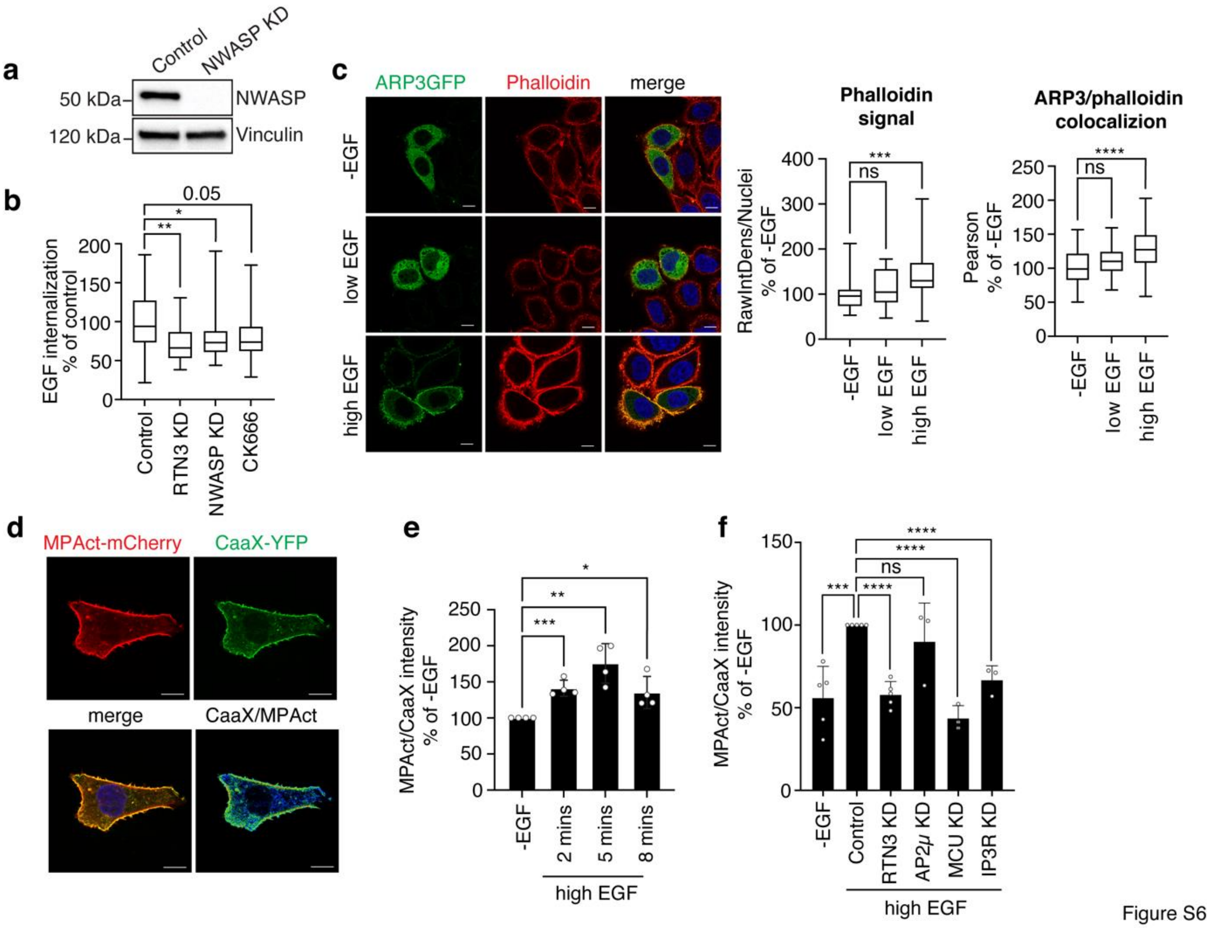

Fig. S6. Regulation of F-actin and ARP3 localization by EGF, and characterization of the cortical actin probe, MPAct, related to Fig. 6. a Efficiency of NWASP KD in HeLa cells analyzed by IB. GAPDH, loading control. MW markers shown on the left. **b** EGF internalization in HeLa cells subjected to the indicated KDs and high dose EGF stimulation (same experiment as in main Fig. 5A). Alexa647-EGF internalization was followed for 5 min at 37°C. Cells were subjected to acid wash treatment prior to fixation to visualize only internalized EGF. Mean integrated fluorescence intensity is reported as a percentage relative to control. RTN3 KD was used as a positive control for NCE inhibition. N=number of cells: Control/N=338 (n=4), RTN3-KD/N=147, NWASP-KD/N=237, CK666/N=250 (n=3). **c** HeLa cells transfected with GFP-ARP3 (green) were stimulated with low or high dose of EGF or left untreated. After fixation, cells were stained with phalloidin-TRITC (red). Left, representative IF images, Blue, DAPI. Bar, 10 µm. Middle, raw integrated density of the phalloidin signal/nuclei is reported as a percentage relative to the -EGF sample. Right, Pearson colocalization coefficient between phalloidin and ARP3 reported as a percentage relative to the -EGF sample. N=number of cells: -EGF/N=196, Low EGF/N=178, High EGF/N=244 (n=3).

d HeLa cells were subjected to MPAct-mCherry and YFP-CaaX co-transfection. A representative IF image split into the different channels, as indicated, is shown. In the merged panel: blue, DAPI. MPAct/CaaX panel: ratiometric analysis is shown in pseudo-colors (rainbow RGB LUT on Image J). Bar, 10 µm. **e** Ratiometric analysis of MPA density in HeLa cells subjected to MPAct-mCherry and YFP-CaaX co-transfection and then stimulated with high EGF dose (100 ng/ml) or left unstimulated for the indicated time points. Mean raw integrated fluorescence intensity ± SD is reported as a percentage relative to -EGF cells. N=number of cells: -EGF/N=164, High EGF/2 mins/N=220, High EGF/5 mins/N=178, High EGF/8 mins/N=157 (n=4). **f** Ratiometric analysis of MPA density in HeLa cells subjected to the indicated KDs, followed by MPAct-mCherry and YFP-CaaX co-transfection and then stimulated with high EGF dose (100 ng/ml). Mean raw integrated fluorescence intensity ±SD is reported as a percentage relative to -EGF cells. N=number of cells: -EGF/N=316, Control/High EGF/N=356, RTN3-KD/N=409 (n=5); AP2µ-KD/N=188, MCU-KD/N=214, IP3R-KD/N=196 (n=3). In the box plots, the lower and upper boundaries of the box are the first and third quartiles, with the median annotated with a line inside the box. The whiskers extend to the maximum and minimum values. All panels, P-value (Each Pair Student's t-test, two-tailed): *, <0.05; **, <0.01; ***, <0.001, ****, <0.0001, ns, not significant; n=biological replicates. Source data are provided as a Source Data file.

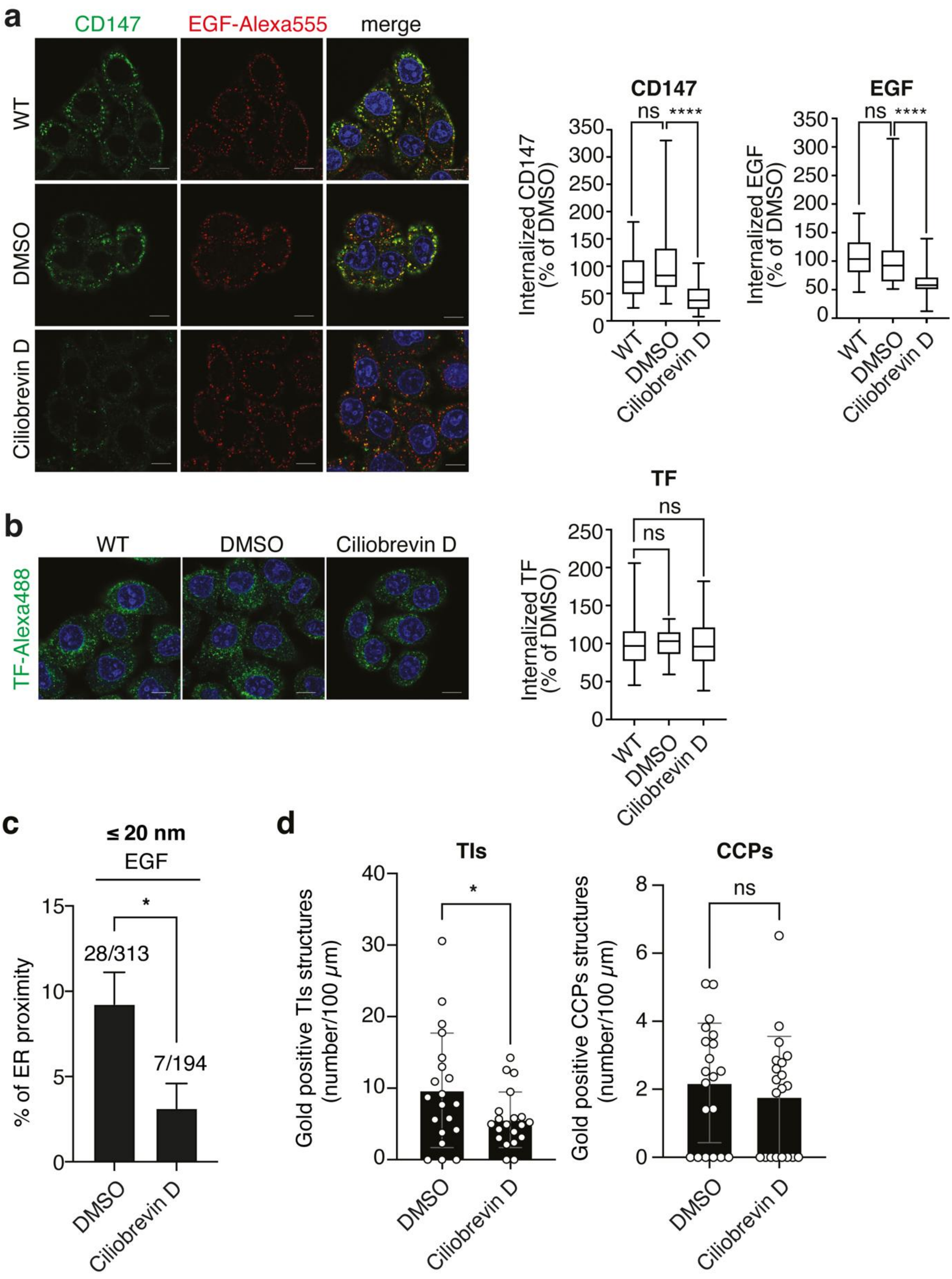

Figure S7

Fig. S7. Characterization of the role of microtubule motor-protein dynein in the regulation of EGFR-NCE. a HeLa cells, treated with Ciliobrevin D (50 µM for 20 min),

DMSO vehicle control or left untreated, were incubated *in vivo* with a specific anti-CD147 antibody for 30 min at 4°C and then stimulated with high dose Alexa555-EGF (100 ng/ml, red) for 8 min at 37°C. Cells were subjected to acid wash treatment prior to fixation to visualize only internalized CD147/EGF. Ciliobrevin D was kept during stimulation. After fixation and permeabilization, cells were incubated with secondary anti-mouse 488 (green). Blue, DAPI. Bar, 10 µm. Left, representative pictures of CD147/EGF internalization are shown. Right, quantification of internalized CD147 (left) and EGF (right). Mean integrated fluorescence intensity is reported as a percentage relative to DMSO. N=number of cells: untreated/N=186, DMSO/N=173, Ciliobrevin D/N=205 (n=3). **b** Transferrin (Tf) internalization in HeLa cells subjected to the indicated treatments. Alexa647-Tf internalization was followed for 8 min at 37°C in HeLa cells treated with Ciliobrevin D (50 µM for 20 min), DMSO vehicle control or left untreated. Mean integrated fluorescence intensity is reported as a percentage relative to control. N, number of cells: untreated/N=187, DMSO/N=189, Ciliobrevin D/N=187 (n=3). **c** Mean frequency of ER proximity with gold EGFR-labelled TIs in HeLa cells either left untreated or pretreated with Ciliobrevin D (50 µM for 5 min), and stimulated with high dose EGF for 5 min (in the presence or not of the drug). Results are expressed as percentage ±SEM. The number of counted structures in contact with the ER is indicated (distance ≤ 20 nm). N= number of cells analyzed: DMSO/N=20, Ciliobrevin D/N=20. **d** Quantification of gold-labeled EGFR-positive TIs in HeLa cells treated as in (c) and stained with RuR. Only RuR-stained structures were counted. Data are expressed as the number of gold-positive structures, normalized to PM profiles of 100-µm length, ±SEM. N, cell profiles analyzed, DMSO/N=20, Ciliobrevin D/N=20. In the box plots, the lower and upper boundaries of the box are the first and third quartiles, with the median annotated with a line inside the box. The whiskers extend to the maximum and minimum values. All panels, P-value (Each Pair Student's t-test, two-tailed): *, <0.05; ****, <0.0001, ns, not significant; n=biological replicates. Source data are provided as a Source Data file.

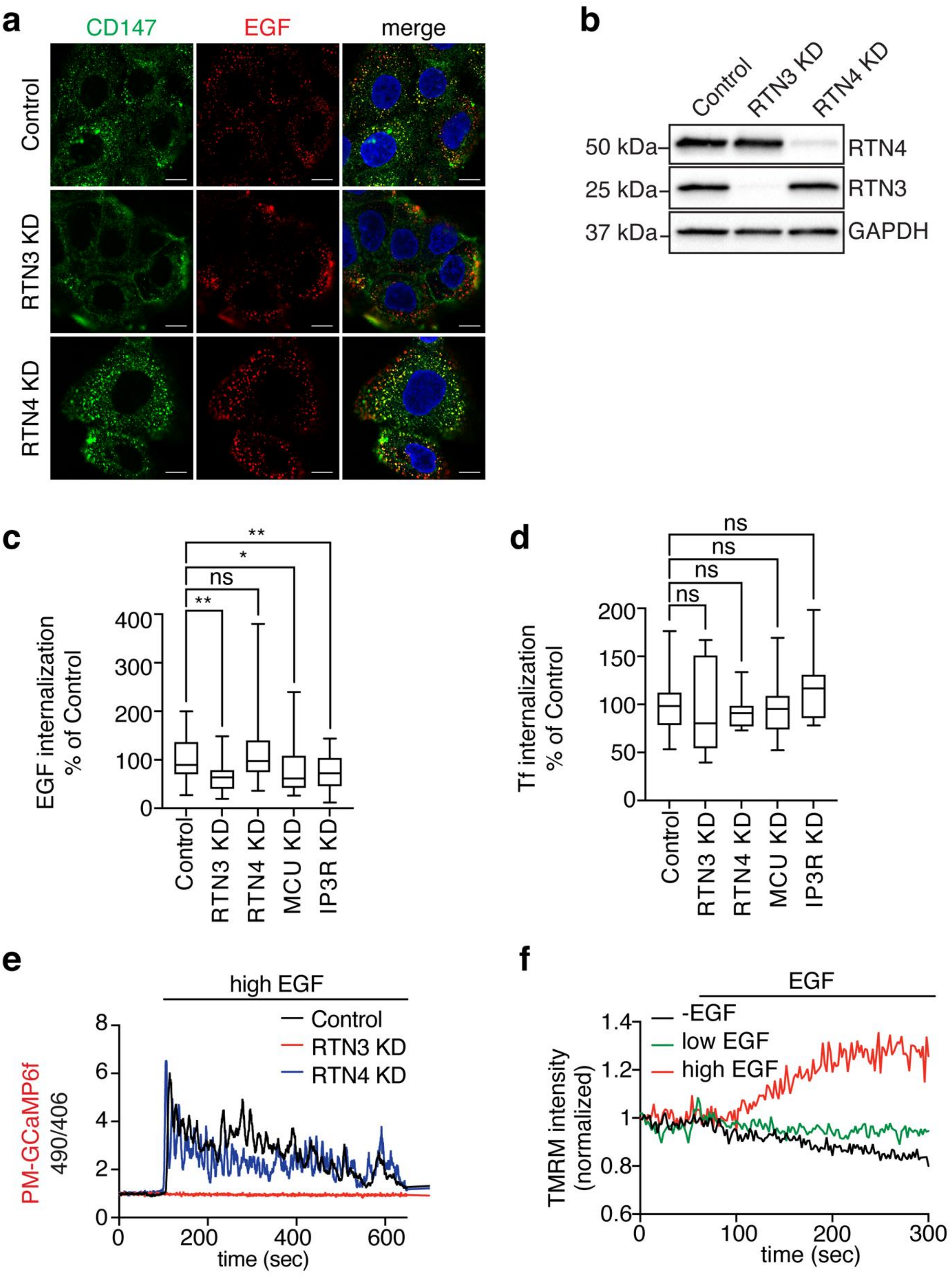

249

Figure S8

Fig. S8. Characterization of EGFR-NCE in the HaCaT cell model system, related to Fig. 7. a CD147 internalization was monitored *in vivo* by IF in HaCaT cells subjected to the indicated KDs or mock transfection and stimulated with high dose EGF. Cells were incubated with an anti-CD147 antibody for 60 min at 4°C, then with an Alexa-488 secondary antibody (green) for 30 min at 4°C before the addition of high dose Alexa647-EGF (red) for 12 min at 37°C. Cells were subjected to acid wash treatment prior to fixation to remove membrane-bound antibodies and analyzed by confocal microscopy. Blue, DAPI. Bar, 10 µm. Quantification is reported in main Fig. 7a. **b** Efficiency of RTN3 and RTN4 KD in HaCaT cells analyzed by IB. GAPDH, loading control. MW markers shown on the left. **c** EGF internalization was monitored in HaCaT cells subjected to the indicated KDs or mock transfection (Control) and stimulated with high dose Alexa647-EGF. Quantification of relative EGF fluorescence intensity is expressed as % of control. N=number of cells: Control/N=352 (n=6), RTN3-KD/N=112 (n=3), RTN4-KD/N=214 (n=5), MCU-KD/N=257 (n=4), IP3R-KD/N=179 (n=3). **d** Alexa647-Tf internalization was followed for 12 min at 37°C in HaCaT cells subjected to the indicated KDs or mock transfection (Control). Mean integrated fluorescence intensity is reported as a percentage relative to control. N=number of cells: Control/N=54, RTN3-KD/N=44, RTN4-KD/N=45, MCU-KD/N=58, IP3R-KD/N=47 (n=1). ns, not significant. **e** HaCaT cells stably expressing PM-GCaMP6f were stimulated with high dose EGF in the presence of transient RTN3 or RTN4 KD or mock control. The Ca^{2+} response was monitored by measuring fluorescence and results are presented as the ratio of the emission at 490/406 nm. Representative single cell curves are reported. Quantitation is shown in main Fig. 7c. **f** HaCaT cells were labeled with TMRM and left unstimulated (- EGF) or stimulated with low or high EGF. Fluorescence TMRM intensity is reported. Quantitation is shown in main Fig. 7d. In the box plots, the lower and upper boundaries of the box are the first and third quartiles, with the median annotated with a line inside the box. The whiskers extend to the maximum and minimum values. All panels, P-value (Each Pair Student's t-test, two-tailed): ***, <0.001; ****, <0.0001, ns, not significant; n=biological replicates. Source data are provided as a Source Data file.

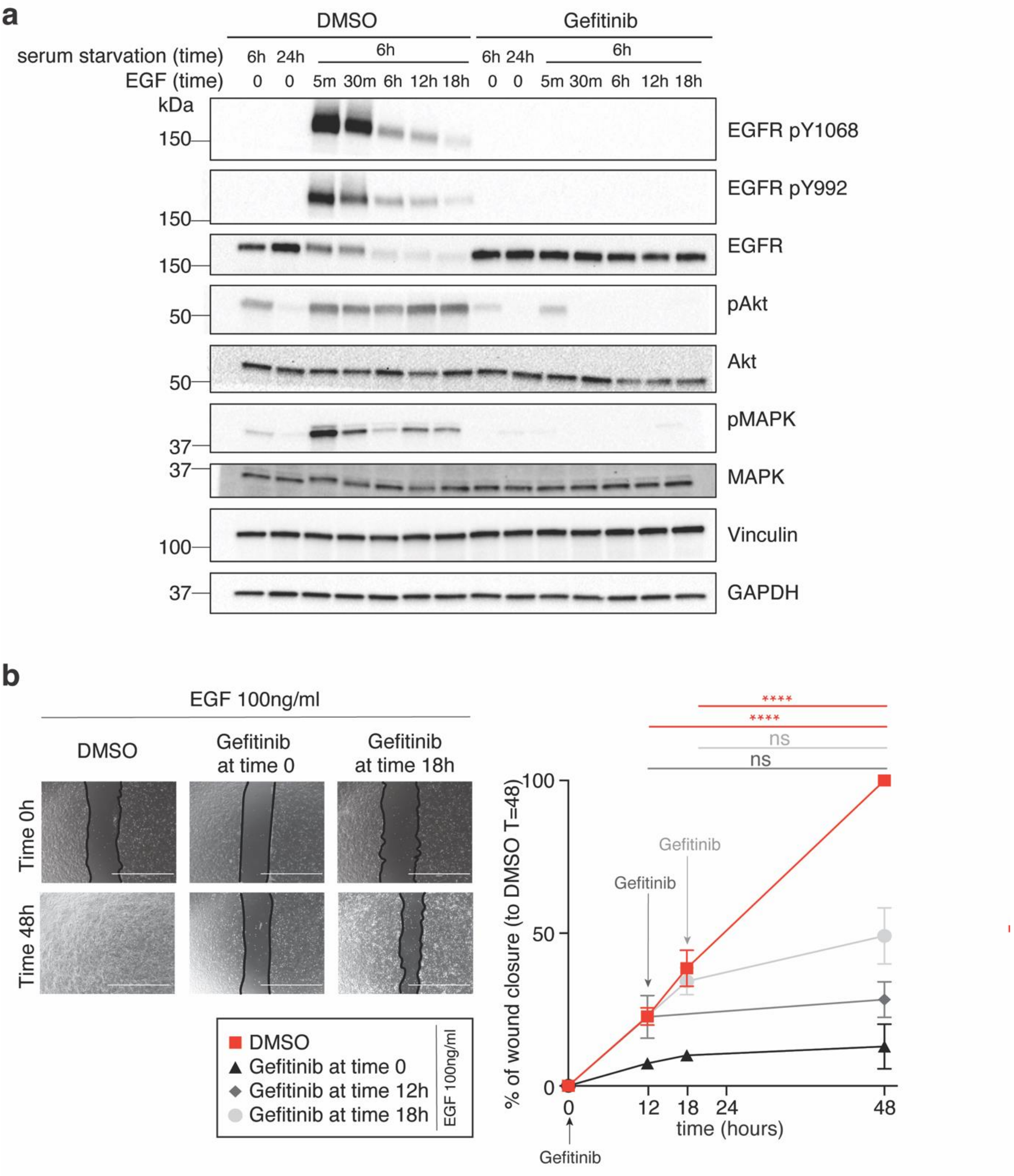

Figure S9

Fig. S9. Long-term EGFR activation sustains signaling and migration in HaCaT cells, related to Fig. 7. **a** HaCaT cells were serum starved for 6h, and then stimulated or not with high-dose EGF (100 ng/ml) for different timepoints in the presence of Gefitinib (5 μM) or vehicle. Lysates were subjected to IB with the indicated antibodies. Vinculin and GAPDH were

used as loading control. MW markers are shown on the left. Samples have been run and blotted on five independent gels. Two representative loading controls (Vinculin, GAPDH) are shown. **b** Wound healing in sub-confluent serum starved HaCaT cells stimulated with high-dose EGF for the indicated time points. Gefitinib (5 µM) or vehicle control were added at different time points after EGF stimulation as indicated. The percentage of closure of the wound was monitored by the acquisition of pictures at EVOS microscope at the different time point. Left, representative images at the indicated time points. Right, the percentage of wound closure ± SD at t=0, 12, 18, 48 hours relative to DMSO+EGF at t=48 is shown (n=3). P-value (Each Pair Student's t-test two-tailed): ****, <0.0001; ns, not significant.

Supplementary Table 1. Antibodies brands and dilutions.

Antibody	Producer	Clone or Epitope	Catalog Number	Usage	Dilution concentration
EGFR 806	Homemade	aa 1172-1186 of human EGFR	REF[5]	WB	33 ng/ml
EGFR pY-1068	Cell Signaling	Tyr 1068, D7A5	# 3777 (XP)	WB	1:1000
EGFR pY-992	Cell Signaling	Tyr 992	#2235	WB	1:1000
SHC	BD	Clone 20	#610878	WB	1:500
pSHC	Cell Signaling	Tyr239/240	#2434	WB	1:500
AKT	Cell Signaling		#9272	WB	1:1000
pAKT	Cell Signaling	Thr308	#9275	WB	1:500
ERK1/2	Sigma	ERK-1, 351-368	M7927	WB	1:5000
pERK1/2	Cell Signaling	Thr202/Tyr204	#9106	WB	1:1000
Vinculin	Sigma	clone hVIN-1	V9131	WB	1:5000
GAPDH	Santa Cruz	6C5	sc-32233	WB	1:3000
gamma-Tubulin	Homemade	Serum		WB	1:1000
EGFR	Genentec	Clone 13A9	13A9	IF/EM	1:2000
HGFR	R&D	Glu25-Thr932	AF276	IF	1:200
CD147	BD	HIM 6	555961	In vivo/IF	1:300

TOMM20	Novus Biologicals		NBP1-81556	CLEM	1:100
Clathrin heavy chain	BD	Clone 23	610499	WB	1:1000
MCU	Sigma		HPA016480	WB	1:500
IP3-R1	Millipore		AB5882	WB	1:1000
IP3-R2	Novus		NB100-2466	WB	1:500
IP3-R3	BD		610312	WB	1:4000
RTN3	Homemade	aa 1-47, common to all RTN3 isoforms		WB	1.5 µg/ml
RTN4	Novus		NB100-5668155	WB	1:500
AP2 p50µ	Transduction BD		m2	WB	1:100
N-WASP	Santa Cruz	H100	sc20770	WB	1:100
FITC-Tyramide	Akoya Biosciences		SKU SAT701001EA	CLEM	1:100
Firefly Luciferase	Invitrogen		PA5-32209	IF<br>WB	1:250<br>1:500

Supplementary Table 2. Oligo brand and sequence.

RTN3 Stealth Invitrogen	5'-CCCUGAAACUCAUUAUUCGUCUCUU-3'
RTN4 Stealth Invitrogen	5’-GGCGCCUCUUCUUAGUUGAUGAUUU-3’
Clathrin Heavy Chain Riboxx	5'-UAAAUUUCCGGGCAAAGAGCCCCC-3'
WASL ON-TARGET Plus Smart Pool Dharmacon	5’-CAGCAGAUCGGAACUGUAU-3’<br>5’-UAGAGAGGGUGCUCAGCUA-3’<br>5’-GGUGUUGCUUGUCUUGUUA-3’<br>5’-CCAGAAAUCACAACAAAUA-3’
IP3-R1 Smart Pool Riboxx	5’-UUAAACGAAAUGCUGCUCCCCC-3’<br>5’-AUAUGUAGAUGUUGUGCCCCC-3’<br>5’-AUAACUAGAUUGGAAGCCCCC-3’
IP3-R2 Smart Pool Riboxx	5’-UUAUUUCUUUCUGAGCAGCCCCC-3’<br>5’-AUUGAUACAAGAAACGGCCCCC-3’<br>5’-AUCUUUAACAUAACAGGCCCCC-3’
IP3-R3 Smart Pool Riboxx	5’-AUUAAGGUAAACUGAGUCCCCC-3’<br>5’-UUAUUCUUGUCAGUCCACGCCCCC-3’

	5’-UAUAGAUGUUAUGGCCCACCCCC-3’
MCU Stealth Invitrogen	5’-GAUCAGGCAUUGUGGAAUAUAAGCU-3’
AP2μ1 Stealth Invitrogen	5’-CAUUGACCCGAAAGGCAUCCACUG-3’
Dynamin 1 Riboxx	5’-UUUCACAAUGGUCUCAAAGCCCCC-3’
Dynamin 2 Riboxx	5’-UGAACUGCAGGAUCAUGUCCCCC-3’
