# Supplementary material for: A tripartite organelle platform links growth factor receptor signaling to mitochondrial metabolism
Source: Nat Commun. 2024 Jun 15;15:5119. doi: 10.1038/s41467-024-49543-z (PMC11180189; doi:10.1038/s41467-024-49543-z)
Supplement: Supplementary file 3 — Description of Additional Supplementary Files [file 41467_2024_49543_MOESM3_ESM.pdf]

Description of Additional Supplementary Files

Supplementary Movie Legends:

Supplementary Movie 1. Tomographic 3D reconstruction showing a tripartite PM-ERmitochondria contact site, related to Fig. 1B. The cortical region of a HeLa cell treated with high dose EGF for 5 min and analyzed by immuno-EM as in Fig. 1A is shown. The spatial proximity between internalizing NCE structures (light grey) containing the cargo CD147 (yellow dots), the ER (blue) and mitochondria (MITO, green) is visible. The ER was automatically recognized through HRP-KDEL staining, while mitochondria were manually segmented.

Supplementary Movie 2. ER tubules interposed between mitochondria and CD147- positive Tis, related to Fig. 1B. Single-tilted tomographic reconstruction (see Materials and Methods) of ER tubules in contact with TIs— internalizing gold-labeled CD147—and mitochondria. Note the contact of ER (pseudocolored in blue) profiles recognized through HRP-KDEL staining with mitochondria (pseudocolor in green) and with CD147- internalizing TI (labeled in gold). Single-tilted tomographic reconstruction, shown in Fig. 1C, was extracted from this movie.

Supplementary Movie 3. EGF-induced Ca2+ oscillations at the PM in HeLa cells detected by PM-GCaMP6f, related to Fig. 2B. Cells treated with low EGF (left), High EGF (center), High EGF + RTN3 KD (right) (5 frame/sec). EGF was added after 45 seconds from the start of the recording. The movie was cut at 300 seconds and accelerated at 75 frame/sec (15x).

Supplementary Movie 4. EGF-induced Ca2+ oscillations inside the mitochondria in HeLa cells detected by mito-GCaMP6m, related to Fig. 2E Cells treated with low EGF (left) or high EGF (right) (5 frame/sec). EGF was added after 45 seconds from the start of the recording. The movie was cut at 300 seconds and accelerated at 75 frame/sec (velocity 15x).

Supplementary Movie 5. EGF-induced increase in $\Delta\Psi m$, related to Fig. 3A. Change in TMRM fluorescence observed in unstimulated HeLa cells (left), or cells stimulated with low (center) or high (right) dose EGF (0.5 frame/sec). EGF was added after 45 seconds from the start of the recording. The movie was accelerated at 50 frame/sec (velocity 100x).

Supplementary Movie 6. High dose EGF promotes collective cell migration in a wound-healing assay, related to Fig. 7E. Timelapse video microscopy showing wound healing in sub-confluent HaCaT cells, unstimulated (left), or treated with low (middle) or high (right) dose EGF for 30 h (1 frame/5 min). The movie was accelerated at 50 frame/sec (velocity 15000x).
