# Supplementary material for: A tripartite organelle platform links growth factor receptor signaling to mitochondrial metabolism
Source: Nat Commun. 2024 Jun 15;15:5119. doi: 10.1038/s41467-024-49543-z (PMC11180189; doi:10.1038/s41467-024-49543-z)
Supplement: Supplementary file 10 — Reporting Summary [file 41467_2024_49543_MOESM10_ESM.pdf]

nature portfolio

Corresponding author(s): Sara Sigismund
Pier Paolo Di Fiore

Last updated by author(s): Mar 22, 2024

Reporting Summary

Nature Portfolio wishes to improve the reproducibility of the work that we publish. This form provides structure for consistency and transparency in reporting. For further information on Nature Portfolio policies, see our Editorial Policies and the Editorial Policy Checklist.

Statistics

For all statistical analyses, confirm that the following items are present in the figure legend, table legend, main text, or Methods section.

n/a	Confirmed	
☐	☒	The exact sample size (*n*) for each experimental group/condition, given as a discrete number and unit of measurement
☐	☒	A statement on whether measurements were taken from distinct samples or whether the same sample was measured repeatedly
☐	☒	The statistical test(s) used AND whether they are one- or two-sided<br>*Only common tests should be described solely by name; describe more complex techniques in the Methods section.*
☐	☒	A description of all covariates tested
☐	☒	A description of any assumptions or corrections, such as tests of normality and adjustment for multiple comparisons
☐	☒	A full description of the statistical parameters including central tendency (e.g. means) or other basic estimates (e.g. regression coefficient) AND variation (e.g. standard deviation) or associated estimates of uncertainty (e.g. confidence intervals)
☐	☒	For null hypothesis testing, the test statistic (e.g. *F*, *t*, *r*) with confidence intervals, effect sizes, degrees of freedom and *P* value noted<br>*Give P values as exact values whenever suitable.*
☒	☐	For Bayesian analysis, information on the choice of priors and Markov chain Monte Carlo settings
☒	☐	For hierarchical and complex designs, identification of the appropriate level for tests and full reporting of outcomes
☐	☒	Estimates of effect sizes (e.g. Cohen's *d*, Pearson's *r*), indicating how they were calculated

Our web collection on statistics for biologists contains articles on many of the points above.

Software and code

Policy information about availability of computer code

Data collection

-EM images were collected with ultramicrotome UC7, Leica microsystem (Vienna, Austria) and Transmission Electron Microscope (TEM) Talos L120C (FEI, Thermo Fisher Scientific).
-Correlative Light Electron Microscopy (CLEM) data were acquired with DeltaVision™ Ultra microscope (GE healthcare).
-Immuno Blotting acquisitions were performed at Bio-Rad ChemiDoc with Image Lab software (Bio-Rad Laboratories).
-Immunofluorescence data were collected at Leica Confocal SP8 Microscope with Las X (Leica).
-PM-Luciferase assay for recording ATP accumulation at the PM and Ca2+-probe Aequorin recording experiments were performed with a custom-made luminescence reader (aequorinometer) equipped with a PMT, a peristaltic pump, a temperature-controlled bath, a photon-counting head and a digitizer counting unit (Hamamatsu) in Prof. Paolo Pinton's laboratory in Ferrara.
-Wound-healing videos were acquired at Nikon IIT-Eclipse-Ti2-Inverted with NIS (Nikon).
- Immunofluorescence data of mitochondria morphology were acquired at Nikon Crest Spinning Disk Confocal Microscope with NIS (Nikon).
-Luminescence counts for detection of intracellular levels of ATP were collected with GloMax® Discover Microplate Reader (Promega).
-TPEF microscopy data were acquired with home-built multimodal optical microscope featuring forward and epi-detected Two-Photon Excited Fluorescence (TPEF and E-TPEF), along with linear transmission light.

Data analysis

-Immuno Blotting analysis was performed with Image Lab software (Bio-Rad Laboratories) and/or Image J.
-Immunofluorescence analysis, EM morphometry, CLEM images, and TPEF image analysis quantification were performed with Image J/Fiji.
-Statistical analysis of IB and IF quantifications, Luciferase/calcium assays were performed with Excel software and/or with GraphPad Prism software (SAS Institute).
-Tomography acquisition software: Tomography 4.0 (FEI, Thermo Fisher Scientific).
-Tomography reconstruction: IMOD software package (Boulder, USA) (Kremer JR, Mastronarde DN, McIntosh JR. Computer visualization of

three-dimensional image data using IMOD. J Struct Biol. 1996 Jan-Feb;116(1):71-6. doi: 10.1006/jsbi.1996.0013. PMID: 8742726.)
-Tomography segmentation: Microscope Image Browser (MIB) (Belevich, Ilya, et al. "Microscopy image browser: a platform for segmentation and analysis of multidimensional datasets." PLoS biology 14.1 (2016): e1002340.)
-3D tomography distance quantification with Matlab.
-CLEM images alignment with ICY software, ec-CLEM plugin.
See Methods for details.

For manuscripts utilizing custom algorithms or software that are central to the research but not yet described in published literature, software must be made available to editors and reviewers. We strongly encourage code deposition in a community repository (e.g. GitHub). See the Nature Portfolio guidelines for submitting code & software for further information.

Data

Policy information about availability of data

All manuscripts must include a data availability statement. This statement should provide the following information, where applicable:

- Accession codes, unique identifiers, or web links for publicly available datasets
- A description of any restrictions on data availability
- For clinical datasets or third party data, please ensure that the statement adheres to our policy

All data are available in the manuscript or in the Supplementary Materials

Research involving human participants, their data, or biological material

Policy information about studies with human participants or human data. See also policy information about sex, gender (identity/presentation), and sexual orientation and race, ethnicity and racism.

Reporting on sex and gender	*Use the terms sex (biological attribute) and gender (shaped by social and cultural circumstances) carefully in order to avoid confusing both terms. Indicate if findings apply to only one sex or gender; describe whether sex and gender were considered in study design; whether sex and/or gender was determined based on self-reporting or assigned and methods used. Provide in the source data disaggregated sex and gender data, where this information has been collected, and if consent has been obtained for sharing of individual-level data; provide overall numbers in this Reporting Summary. Please state if this information has not been collected. Report sex- and gender-based analyses where performed, justify reasons for lack of sex- and gender-based analysis.*
Reporting on race, ethnicity, or other socially relevant groupings	*Please specify the socially constructed or socially relevant categorization variable(s) used in your manuscript and explain why they were used. Please note that such variables should not be used as proxies for other socially constructed/relevant variables (for example, race or ethnicity should not be used as a proxy for socioeconomic status). Provide clear definitions of the relevant terms used, how they were provided (by the participants/respondents, the researchers, or third parties), and the method(s) used to classify people into the different categories (e.g. self-report, census or administrative data, social media data, etc.) Please provide details about how you controlled for confounding variables in your analyses.*
Population characteristics	*Describe the covariate-relevant population characteristics of the human research participants (e.g. age, genotypic information, past and current diagnosis and treatment categories). If you filled out the behavioural & social sciences study design questions and have nothing to add here, write "See above."*
Recruitment	*Describe how participants were recruited. Outline any potential self-selection bias or other biases that may be present and how these are likely to impact results.*
Ethics oversight	*Identify the organization(s) that approved the study protocol.*

Note that full information on the approval of the study protocol must also be provided in the manuscript.

Field-specific reporting

Please select the one below that is the best fit for your research. If you are not sure, read the appropriate sections before making your selection.

☒ Life sciences ☐ Behavioural & social sciences ☐ Ecological, evolutionary & environmental sciences

For a reference copy of the document with all sections, see nature.com/documents/nr-reporting-summary-flat.pdf

Life sciences study design

All studies must disclose on these points even when the disclosure is negative.

Sample size	No sample size calculations were performed; the number of experiments (n) is indicated in each figure legend.
Data exclusions	No data were excluded
Replication	The number of experiments replicated (n) is indicated in each figure legend

Randomization	Samples were randomly assigned.
Blinding	N/A

Reporting for specific materials, systems and methods

We require information from authors about some types of materials, experimental systems and methods used in many studies. Here, indicate whether each material, system or method listed is relevant to your study. If you are not sure if a list item applies to your research, read the appropriate section before selecting a response.

Materials & experimental systems

n/a	Involved in the study
☐	☒ Antibodies
☐	☒ Eukaryotic cell lines
☒	☐ Palaeontology and archaeology
☒	☐ Animals and other organisms
☒	☐ Clinical data
☒	☐ Dual use research of concern
☒	☐ Plants

Methods

n/a	Involved in the study
☒	☐ ChIP-seq
☒	☐ Flow cytometry
☒	☐ MRI-based neuroimaging

Antibodies

Antibodies used

Antibodies used:
Primary Ab:
-homemade rabbit polyclonal antibody anti-EGFR806 (epitope: aa 1172-1186, Homo sapiens) The concentration used is 33 ng/ml for IB.
-homemade rabbit polyclonal antibody anti-RTN3 (epitope: aa 1-47, Homo sapiens, common to all RTN3 isoforms). The concentration used is 1.5 µg/ml for IB.
-homemade rat polyclonal antibody anti-gamma-Tubulin. The Dilution used is 1:1000 for IB.
-anti-vinculin, Sigma, clone hVIN-1, V9131. 1:5000 for IB.
-anti-tubulin, Sigma, clone DM1A, T9026. 1:5000 for IB.
-anti-GAPDH, Santa Cruz, clone 6D5, sc-32233. 1:3000 for IB.
-anti-EGFR, Genentec, clone 13A9. 1:2000 for IB/IF.
-anti-clathrin heavy chain, BD Bioscience, clone 23, 610499. 1:1000 for IB.
-anti-pY 1068 EGFR, Cell Signaling, epitope Tyr 1068, D7A5, #3777 (XP). 1:1000 for IB.
-anti-pY 992 EGFR, Cell Signaling, epitope Tyr 992, #2235 (XP). 1:1000 for IB.
-anti-SHC, BD, clone 20, #610878. 1:500 for IB.
-anti-pSHC, Cell Signaling, epitope Tyr239/240, #2434. 1:500 for IB.
-AKT, Cell Signaling, #9272. 1:1000 for IB.
-pAKT, Cell Signaling, epitope Thr308, #9275. 1:500 for IB.
-ERK1/2, Sigma, epitope ERK-1, 351-368, M7927. 1:5000 for IB.
-pERK1/2, Cell Signaling, epitope Thr202/Tyr204, #9106. 1:1000 for IB.
-HGFR, R&D, epitope Glu25-Thr932, AF276. 1:200 for IB.
-CD147, BD, clone HIM 6, 555961. 1:300 for in vivo IF.
-TOMM20, Novus Biologicals, NBP1-81556. 1:100 for CLEM.
-MCU, Sigma, HPA016480. 1:500 for IB.
-IP3-R1, Millipore, AB5882. 1:1000 for IB.
-IP3-R2, Novus , NB100-2466. 1:500 for IB.
-IP3-R3, BD, 610312. 1:4000 for IF, I:100 for PLA.
-RTN4, Novus, NB100-5668155. 1:500 for IB.
-AP2 p50µ, Transduction BD, Clone 31/AP50 (RUO), 611350. 1:100 for IB.
-N-WASP, Santa Cruz, clone H100, sc20770. 1:100 for IB.
-FITC-Tyramide, Akoya Biosciences, SKU SAT701001EA. 1:100 for CLEM.
-Firefly Luciferase, Invitrogen, PA5-32209. 1:250 for IF, 1:500 for IB.

Secondary Ab:
-anti-rabbit IgG HRP-linked, Cell Signaling, 7074. 1:2000 for IB.
-anti-mouse IgG HRP-linked, Cell Signaling, 7076. 1:2000 for IB.
-anti-rat IgG HRP-linked, Cell Signaling, 7077. 1:2000 for IB.

-Alexa Fluor 488 AffiniPure Donkey anti-Mouse IgG (H+L), Jackson ImmunoResearch, 715-545-150. 1:200 for IF.
-Alexa Fluor 488 AffiniPure Donkey anti-Rabbit IgG (H+L), Jackson ImmunoResearch, 715-545-152. 1:200 for IF.
-Alexa Fluor 647 AffiniPure Donkey anti-Mouse IgG (H+L), Jackson ImmunoResearch, 715-605-150. 1:200 for IF.
-Alexa Fluor 647 AffiniPure Donkey anti-Mouse IgG (H+L), Jackson ImmunoResearch, 715-605-152. 1:200 for IF.
-Cy3 donkey anti rabbit IgG, Jackson ImmunoResearch,715-165-152. 1:200 for IF.
-Cy3 donkey anti mouse IgG, Jackson ImmunoResearch, 715-165-150. 1:200 for IF

Validation	Homemade antibodies (anti-EGFR806 and anti-RTN3), anti-clathrin heavy chain, anti-MCU, anti-IP3-R1, anti-IP3-R2, anti-IP3-R3, anti-RTN4, anti-AP2 p50µ and anti-N-WASP were validated in-house through RNA interference and IB and/or IF.<br><br>Validation statements available from manufacturers:<br>-anti-vinculin: https://www.sigmaaldrich.com/catalog/product/SIGMA/V9131?lang=it&region=IT<br>-anti- clathrin heavy chain: https://www.bdbiosciences.com/eu/reagents/research/antibodies-buffers/cell-biology-reagents/cell-biology-antibodies/purified-mouse-anti-clathrin-heavy-chain-23clathrin-heavy-chain/p/610499<br>-anti-tubulin: https://www.sigmaaldrich.com/IT/it/product/sigma/t4026<br>-anti-GAPDH: https://www.scbt.com/it/p/gapdh-antibody-6c5<br>-anti-pY 1068 EGFR: https://www.cellsignal.com/products/primary-antibodies/phospho-egf-receptor-tyr1068-d7a5-xp-rabbit-mab/3777<br>-anti-pY 992 EGFR: https://www.cellsignal.com/products/primary-antibodies/phospho-egf-receptor-tyr992-antibody/2235<br>-anti-EGFR, clone 13A9. It is a mouse monoclonal raised against the ectodomain of EGFR that does not compete with EGF binding. A gift from Genentech.<br>-anti-SHC: https://www.bdbiosciences.com/en-at/products/reagents/microscopy-imaging-reagents/immunofluorescence-reagents/purified-mouse-anti-shc.610878<br>-anti-pSHC: https://www.cellsignal.com/products/primary-antibodies/phospho-shc-tyr239-240-antibody/2434<br>-anti-AKT: https://www.cellsignal.com/products/primary-antibodies/akt-antibody/9272<br>-anti-pAKT: https://www.cellsignal.com/products/primary-antibodies/phospho-akt-thr308-antibody/9275<br>-anti-ERK1/2: https://www.sigmaaldrich.com/IT/it/product/sigma/m7802<br>-anti-pERK1/2: https://www.cellsignal.com/products/primary-antibodies/phospho-p44-42-mapk-erk1-2-thr202-tyr204-e10-mouse-mab/9106?productId=6956&Ns=productCitationsCount%7C1&N=4294956287&Nrpp=100&fromPage=plp<br>-anti-HGFR, R&D: https://www.rndsystems.com/products/human-hgfr-c-met-antibody_af276<br>-anti-CD147: https://www.bdbiosciences.com/en-pt/products/reagents/flow-cytometry-reagents/research-reagents/single-color-antibodies-ruo/purified-mouse-anti-human-cd147.555961<br>-anti-TOMM20: https://www.novusbio.com/products/tomm20-antibody_nbp1-81556<br>-anti-MCU: https://www.sigmaaldrich.com/IT/it/product/sigma/hpa016480?utm_source=google&utm_medium=cpc&utm_campaign=20849242488&utm_content=156828976219&gclid=Cj0KCQjw2PSvBhDjARIsAKc2cgNYbuUINol5cKR_mElXtqfJCUdnSPB4fqHvmXDDaIWjARSEIa2JGcsaAvWJEALw_wcB<br>-anti-IP3-R1: https://www.merckmillipore.com/IT/it/product/Anti-IP3-Receptor-1-Antibody,MM_NF-AB5882-200UL<br>-anti-IP3-R2: https://www.novusbio.com/products/itpr2-antibody_nb100-2466<br>-anti-IP3-R3: https://www.bdbiosciences.com/en-ch/products/reagents/microscopy-imaging-reagents/immunofluorescence-reagents/purified-mouse-anti-ip3r-3.610312<br>-anti-RTN4: https://www.novusbio.com/products/nogo-antibody_nb100-56681<br>-anti-AP2 p50µ: https://www.bdbiosciences.com/en-it/products/reagents/microscopy-imaging-reagents/immunofluorescence-reagents/purified-mouse-anti-ap50.611350<br>-anti-N-WASP: https://www.scbt.com/it/p/n-wasp-antibody-h-100<br>-FITC-Tyramide, Akoya Biosciences: https://my.akoyabio.com/ccrz__ProductDetails?sku=SAT701001EA&cclcl=en_US<br>-anti-Firefly Luciferase: https://www.thermofisher.com/antibody/product/Firefly-luciferase-Antibody-Polyclonal/PA5-32209<br><br>Secondary Ab:<br>-Anti-IgG RABBIT HRP: https://www.cellsignal.com/products/secondary-antibodies/anti-rabbit-igg-hrp-linked-antibody/7074<br>-Anti-IgG MOUSE HRP: https://www.cellsignal.com/products/secondary-antibodies/anti-mouse-igg-hrp-linked-antibody/7076<br>-Anti-IgG RAT HRP: https://www.cellsignal.com/products/secondary-antibodies/anti-rat-igg-hrp-linked-antibody/7077<br><br>- Alexa Fluor 488 AffiniPure Donkey anti-Mouse IgG (H+L), Jackson ImmunoResearch: https://www.jacksonimmuno.com/catalog/products/715-545-150<br>-Alexa Fluor 488 AffiniPure Donkey anti-Rabbit IgG (H+L), Jackson ImmunoResearch: https://www.jacksonimmuno.com/catalog/products/711-545-152<br>-Alexa Fluor 647 AffiniPure Donkey anti-Mouse IgG (H+L), Jackson ImmunoResearch: https://www.jacksonimmuno.com/catalog/products/715-605-150<br>-Alexa Fluor 647 AffiniPure Donkey anti-Mouse IgG (H+L), Jackson ImmunoResearch: https://jacksonimmuno.com/catalog/products/711-605-152<br>-Cy3 donkey anti rabbit IgG, https://www.jacksonimmuno.com/catalog/products/711-165-152<br>-Cy3 donkey anti mouse IgG, https://www.jacksonimmuno.com/catalog/products/715-165-150

Eukaryotic cell lines

Policy information about cell lines and Sex and Gender in Research

Cell line source(s)	HaCaT (human immortalized keratinocytes) cell line was purchased from CLS (Cell Lines Service, 300493). HeLa (human cervix epithelial cells) is an isolate from IEO Institute. HeLa Oslo cells were kindly provided by Prof. IH Madshus (University of Oslo, Norway). HeLa and HeLa OSLO were previously described in Sigismund S., et al., EMBO J 2013.
Authentication	All human cell lines were authenticated at each batch freezing by STR profiling (StemElite ID System, Promega).
Mycoplasma contamination	All human cell lines were tested for mycoplasma by PCR (Uphoff and Drexler, 2002) and biochemical assay (MycoAlert, Lonza).

Commonly misidentified lines
(See ICLAC register)

NO

Plants

Seed stocks

Report on the source of all seed stocks or other plant material used. If applicable, state the seed stock centre and catalogue number. If plant specimens were collected from the field, describe the collection location, date and sampling procedures.

Novel plant genotypes

Describe the methods by which all novel plant genotypes were produced. This includes those generated by transgenic approaches, gene editing, chemical/radiation-based mutagenesis and hybridization. For transgenic lines, describe the transformation method, the number of independent lines analyzed and the generation upon which experiments were performed. For gene-edited lines, describe the editor used, the endogenous sequence targeted for editing, the targeting guide RNA sequence (if applicable) and how the editor was applied.

Authentication

Describe any authentication procedures for each seed stock used or novel genotype generated. Describe any experiments used to assess the effect of a mutation and, where applicable, how potential secondary effects (e.g. second site T-DNA insertions, mosiacism, off-target gene editing) were examined.
